# Parental decision making involvement and decisional conflict: a descriptive study

**DOI:** 10.1186/s12887-017-0899-4

**Published:** 2017-06-13

**Authors:** Laura Boland, Jennifer Kryworuchko, Anton Saarimaki, Margaret L. Lawson

**Affiliations:** 1 University of Ottawa, Faculty of Health Sciences, Population Health, 125 University Street, room 232, Ottawa, ON K1N 6N5 Canada; 2 University of Saskatchewan College of Nursing Health Sciences, E-4220, 104 Clinic Place, Saskatoon, S7N 5E5 SK Canada; 3 Ottawa Hospital Research Institute & University of Ottawa, 501 Smyth Road, Box 711, Ottawa, ON K2G 0Y1 Canada; 4 Family Decision Services, CHEO Research Institute, Children’s Hospital of Eastern Ontario, University of Ottawa, 401 Smyth Road, Ottawa, K1H 8L1 ON Canada; 5 Present address: School of Nursing, University of British Columbia, Vancouver, Canada

**Keywords:** Pediatrics, Parents, Decisional conflict, Shared decision making, Family centered care

## Abstract

**Background:**

Decisional conflict is a state of uncertainty about the best treatment option among competing alternatives and is common among adult patients who are inadequately involved in the health decision making process. In pediatrics, research shows that many parents are insufficiently involved in decisions about their child’s health. However, little is known about parents’ experience of decisional conflict. We explored parents’ perceived decision making involvement and its association with parents’ decisional conflict.

**Method:**

We conducted a descriptive survey study in a pediatric tertiary care hospital. Our survey was guided by validated decisional conflict screening items (i.e., the SURE test). We administered the survey to eligible parents after an ambulatory care or emergency department consultation for their child.

**Results:**

Four hundred twenty-nine respondents were included in the analysis. Forty-eight percent of parents reported not being offered treatment options and 23% screened positive for decisional conflict. Parents who reported being offered options experienced less decisional conflict than parents who reported not being offered options (5% vs. 42%, *p* < 0.001). Further, parents with options were more likely to: feel sure about the decision (RR 1.08, 95% CI 1.02–1.15); understand the information (RR 1.92, 95% CI 1.63–2.28); be clear about the risks and benefits (RR 1.12, 95% CI 1.05–1.20); and, have sufficient support and advice to make a choice (RR 1.07, 95% CI 1.03–1.11).

**Conclusion:**

Many parents in our sample experienced decisional conflict after their clinical consultation. Involving parents in the decision making process might reduce their risk of decisional conflict. Evidence based interventions that support parent decision making involvement, such as shared decision making, should be evaluated and implemented in pediatrics as a strategy to reduce parents’ decisional conflict.

**Electronic supplementary material:**

The online version of this article (doi:10.1186/s12887-017-0899-4) contains supplementary material, which is available to authorized users.

## Background

Family centered care, evidence-based clinical decision making, and patient engagement require that patient preferences guide the decision making process [1–3]. Patient engagement for health decisions is optimized when patient and family preferences, medical evidence, and clinical judgment inform the treatment plan [3, 4]. In pediatrics, health legislation and policies typically require parent, legal guardian, or surrogate decision maker involvement in treatment decisions when the child has not reached the legal age of majority, is unable to make health decisions independently, or requires support to provide consent or assent [5, 6]. Parents want an active role in health decision making for their child; however, integrated and narrative reviews show that many parents are inadequately involved in decisions about their child’s health [7, 8].

The Ottawa Decision Support Framework is an evidence-based theoretical framework for guiding patient health decision making [9]. It asserts that individuals’ decisional needs affect the quality of a decision (i.e. informed, values-based choices), which in turn affects behavior (e.g. delaying or changing decisions), health outcomes, emotions (e.g. regret, blame), and use of health services. When adult patients are poorly involved in health decisions they are more likely to experience decisional conflict, a state of personal uncertainty [10]. Like adult patients, parents who experience unresolved decisional conflict might encounter undesired outcomes, such as delaying the decision or blaming the healthcare team for undesired outcomes [11, 12]. For example, several cohort studies have identified a positive association between parental decisional conflict and decision regret when making surgical decisions [13, 14].

Decisional conflict about treatment choices is more prevalent among those who are uninformed, unclear about the risks and benefits, uncertain about their values and preferences, or feel inadequately supported [9, 10]. Presentation of treatment options is a necessary first step to informing and involving patients and family members in health decision making. Knowledge of options is also necessary for making informed decisions, ensuring patient and family members’ preferences guide the decision making process, and obtaining informed consent [15, 16]. In fact, failing to present available options is a key barrier to involving patients and families in health decision making [17–19].

Shared decision making is an evidenced-based health decision making approach that promotes partnership between healthcare professionals, patients, and parents [20]. By exchanging information about the medical evidence (options, risks, and benefits) and the family’s preferences and values, healthcare professionals, patients, and parents can deliberate to determine the best treatment plan [15]. Shared decision making interventions have been shown in a Cochrane review to improve involvement and reduce decisional conflict among adult patients [16]. A meta-analysis of nine studies that evaluated pediatric shared decision making interventions also showed a significant reduction in decisional conflict for parents exposed to the intervention [21]. As such, involving parents in the decision making process has the potential to improve decisional outcomes, such as decisional conflict.

Contextual factors, such as condition, severity of the illness, and the quality of interactions are known to influence decisional conflict and the decision making process [22–24]. For example, 6% of women considering prenatal screening for Down syndrome experienced decisional conflict [25]. In contrast, 28% of parents considering hypospadias repair for their son and 33% of parents considering otoplasty experienced decisional conflict [26, 27]. Consideration of context is important in order to better understand parents’ experience of the decision making process and decisional conflict [22].

Little is known about the relationship between parents’ perceived decision making involvement and their experience of decisional conflict, or the influence of context (e.g., clinical setting) on decisional conflict. As such, this study aimed to explore:

1. Whether parents perceived being offered treatment options after discussing a decision about their child’s health with a healthcare professional.
2. Whether parents experienced decisional conflict after discussing a decision about their child’s health with a healthcare professional.
3. The relationship between parents’ perception of being offered treatment options and decisional conflict, after discussing a decision about their child’s health with a healthcare professional.
4. Whether the proportion of parents’ who experience decisional conflict is different across clinical settings.
5. Whether parents perceived that the healthcare team made efforts to involve them in the decision making process.

## Methods

### Design

We conducted a descriptive study using a self-administered survey. Our study was approved by the Children’s Hospital of Eastern Ontario’s Research Ethics Board (approval number 10/91X). We followed STROBE reporting guidelines.

### Setting and participants

We administered the survey in a tertiary academic pediatric hospital with more than 2500 physicians, nurses, and staff serving a population of approximately 600,000 children aged 18 years or younger. The survey was conducted in ambulatory care clinics (medical, surgical and mental health) and the emergency department. These clinical areas were purposefully selected for two reasons: (a) due to their wide cross-section of patient ages, health status, and high patient volume (approximately 170,845 ambulatory care clinic visits and 66,050 emergency department visits annually) [28]; and, (b) to inform the implementation of shared decision making interventions within these clinical areas of our hospital by assessing parents’ decisional needs. These shared decision making interventions are described elsewhere [29–31].

A convenience sample of parents, legal guardians, and temporary surrogate decision makers (collectively referred to as ‘parents’) were recruited. Parents were eligible if they self-identified that a decision about their child’s health had been discussed during the clinical encounter immediately preceding the survey. Parents were ineligible if their child was in crisis or admitted to the hospital, or if the parent was considered by clinical staff too distressed to be informed of the study.

### Survey

Our research team developed the survey based on the Ottawa Decision Support Framework [9] (Additional file 1), which shows that decision making can be adversely affected by various factors, including: type of decision, inadequate knowledge of the options, decisional conflict, unclear values related to the outcome of the options, and insufficient support. The first survey item asked parents to identify the decision that was discussed during the preceding clinical encounter. Consistent with our research questions, parents who perceived that a decision was not discussed were advised to discontinue the survey and were excluded from the analysis. The following items asked parents which healthcare professional(s) were involved in the discussion (1 item); and, whether they were asked to consider more than one option (1 item). Next, we screened parents for decisional conflict using the Decisional Conflict Scale (4-item SURE test version) [25].

The Decisional Conflict Scale is a validated research measurement instrument that assesses modifiable decisional conflict factors, such as patient knowledge, values, and support. The scale discriminates between those who make or delay decisions, is correlated with knowledge, decisional regret and discontinuation, and has acceptable internal reliability of >0.84 among parents making decisions about their child with life threatening illnesses [32]. The SURE version of the Decisional Conflict Scale was designed to quickly identify patients in clinical settings with clinically significant decisional conflict [25]. Individuals who score less than perfect (i.e., < 4/4) on the SURE test screen positive for clinically significant decisional conflict. The four items include: (a) certainty about the decision; (b) knowledge of the risks and benefits of each option; (c) personal values and preferences; and, (d) support and advice (Table 1). The SURE test has acceptable internal consistency (KR-20 coefficient of 0.70) and diagnostic validity among adult patients and parents making decisions about their child’s health with 90.1% accuracy, 94% sensitivity, and 90% specificity in primary care settings with low prevalence of decisional conflict [33].

**Table 1:** The SURE test

SURE Acronym	Items	Yes [1]	No [0]
Sure of myself	Do you feel SURE about the best choice for you?		
Understand information	Do you know the benefits and risks of each option?		
Risk-benefit ratio	Are you clear about which benefits and risks matter most to you?		
Encouragement	Do you have enough support and advice to make a choice?		

Individuals who answer “no” to one or more questions screen positive for decisional conflict

The SURE Test © O’Connor and Légaré, 2008; this copyrighted table is being reprinted with permission from the developers

Respondents were then asked to rate the healthcare team’s efforts using a 5-point scale (1 = very poor to 5 = very good) regarding: involving them in the discussion (1 item); encouraging them to share information about their child (1 item); and, encouraging them to provide suggestions for their child’s care (1 item). The remaining items asked the age of the child involved in the health decision (1 item) and who was filling out the survey (1 item).

The survey was reviewed for face validity by experts and piloted with a parent sample. First, using an iterative process, the survey was presented to pediatricians (*n* = 4) for feedback. Second, the survey was reviewed and accepted by a panel of experts in shared decision making (*n* = 3). Finally, the survey was piloted with a convenience sample of 16 parents across four ambulatory clinics. No changes to the survey or data collection procedures were indicated based on pilot feedback. All participating ambulatory care clinic and emergency department chiefs of staff approved this study. Data were collected over the course of a 1-month period, with approximately 55 h of data collection in each clinical setting.

### Survey procedures

Survey administration procedures were adapted to the ambulatory care clinics’ and emergency department’s processes of care. In ambulatory care clinics, a receptionist introduced the survey to parents during patient registration. Interested parents were asked to approach the research assistant, stationed in the waiting area, after their clinic visit. Our emergency department has research volunteers trained to conduct studies. Upon registration, the receptionist asked parents if they were willing to be approached by a research volunteer to complete a survey after their visit.

Once approached, the research assistant or trained research volunteer used a written script to describe the purpose of the survey and to advise potential participants that the survey was anonymous, voluntary, and that their responses would be kept confidential. Parents were given the opportunity to ask questions. Interested parents were then advised that if they chose to fill out the survey, that would mean they were providing consent to participate. Participants were provided a clipboard, pen, and one copy of the survey. Depending on the resources available in each clinic, participants were invited to complete the survey in an examination room, a quiet station set up in the hallway outside the clinic, or in a reserved seat in the clinical waiting area. The survey was administered immediately after the clinical encounter to minimize recall bias. Upon completion, participants folded their survey and placed it in a clearly identified box next to the research assistant or volunteer. As compensation for their time, participants were invited to enter their name into a draw to win a ticket for our hospital’s annual Dream of a Lifetime charity (ticket value $100). Failure to complete the survey did not disqualify participants from entering the draw. The research assistant or volunteer obtained the number of patients who had registered for clinic/emergency visits during the time the survey was administered to facilitate response rate calculation.

### Analysis

Raw data were manually entered into a Microsoft Excel spreadsheet (Microsoft Corporation, Redmond, WA, USA) and transferred to Statistical Analysis Software for Windows (version 9.4: SAS Institute, Cary, NC, USA). Descriptive analyses for all items were calculated and summarized as percentages. Chi-squared tests were used to explore differences between respondent type, decision discussed, and healthcare professional involved. Parents were also categorized according to whether they reported being offered treatment options, then analyzed across SURE test items using chi-squared tests and risk ratios with 95% confidence intervals. Parents’ ratings of the healthcare team’s efforts to involve them in the decision making process were also categorized according to whether they perceived being provided options and compared using a non-parametric Wilcoxon test. Comparative sub-analyses for ambulatory care and emergency department comparisons were conducted on an exploratory basis using a chi-squared test. All *p* values were two-sided with statistical significance set at 0.05.

## Results

Of the 1156 patients registered for a clinical encounter in ambulatory care or the emergency department during the data collection period, 480 parents completed a survey. Our most conservative response rate calculation is 41.5% (480/1156) given that the denominator included clinic registrants who were potentially ineligible for the survey (e.g., youth attended the consultation without a parent, child was admitted to hospital, or family was too distressed to participate). Fifty-one parents reported that a decision was not discussed during their consultation; the remaining 429 surveys were included in the analysis. ‬‬‬‬‬‬‬‬‬‬‬‬‬‬‬‬‬‬‬‬‬‬

Of 429 parents, ‬153 (36%) visited an ambulatory care clinic and 276 (64%) visited the emergency department. The average clinical setting response rate was 32% for ambulatory care and 51% for the emergency department. There were no significant differences between parents’ responses from ambulatory care clinics and the emergency department with respect to respondent type, healthcare professional involved, type of decision discussed, and age of the child involved. Most respondents were mothers (63%), followed by fathers (29%), and others (6%) (e.g., grandparent, step-parent, and foster parent). Although not mutually exclusive, health professionals involved included staff physicians (74%), residents or fellows (24%), nurse practitioners (20%), medical students (10%), and others (e.g., allied health professional) (6%). The most common types of decisions discussed were tests (56%), medications (54%), follow-up (e.g., referral to specialist) (44%), behavioral intervention (13%), and surgery (8%). The mean age of the child whose health was discussed was 6 years (*SD* = 5.9; range 0–18 years).

### Decisional conflict

**Fig. 1:**
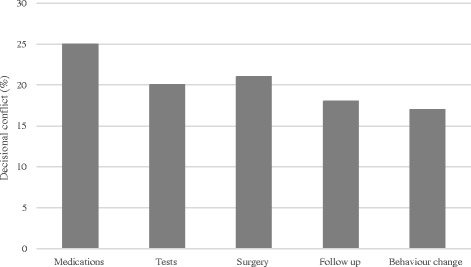
Percentage of parents who screened positive for decisional conflict based on decision type

Of the 412 parents who responded to the SURE test, 23% screened positive for decisional conflict. Of these, 7% were uncertain about the best choice, 23% reported incomplete knowledge of the benefits and harms of the options, 8% lacked clarity of the risks and benefits that matter most to them, and 3% had insufficient support and advice to make a decision. More parents from the emergency department screened positive for decisional conflict compared to ambulatory clinics (24% vs. 16%; *p* = 0.04). However, there were no statistically significant differences between clinical settings on participants’ specific SURE test responses: certainty about the decision (93% vs. 92%, *p* = 0.74), understanding the information (82% vs. 75%, *p* = 0.13), clarity about the risks and benefits (95% vs 91%, *p* = 0.13), and support and advice (96% vs. 97%, *p* = 0.70). Overall, there was no significant difference in decisional conflict based on decision type (*p* = 0.16) (Fig. 1).

Two hundred and seven parents (52%) reported being offered more than one option. There was no difference in the proportion of parents who reported being offered options between the emergency department and ambulatory care clinics (52% vs. 53%, *p* = 0.84). Parents who reported discussing options experienced less decisional conflict than those who perceived no options were provided (5% vs. 42%, *p* < 0.001). Further, parents with options were more likely to: feel sure about their choice; understand the information; be clear about the risks and benefits that mattered most to them; and, have sufficient support and advice to make a choice (Table 2).

**Table 2:** Parents’ perception of provision of options and indicators of decisional conflict

Decisional Conflict Indicators (SURE test)	Reported no options given	Reported options given	Risk ratio	95% CI	*P* value
	Parents *n* (%)	Parents *n* (%)			
Sure of myself	156/176 (89%)	190/198 (96%)	1.08	1.02–1.15	0.007
Understand information	71/142 (50%)	188/195 (96%)	1.92	1.63–2.28	0.0002
Risks benefits ratio	146/169 (86%)	195/201 (97%)	1.12	1.05–1.20	<0.0001
Encouragement	164/176 (93%)	203/204 (100%)	1.07	1.03–1.11	0.0007

### Ratings of healthcare professionals

Parents rated the healthcare team’s efforts to involve them in the decision making process most favorably (‘good’ and ‘very good’ responses combined) for: involving them in the discussion (97% positive ratings; *n* = 412), encouraging them to share information about their child (94% positive ratings; *n* = 386), and eliciting parents’ suggestions for their child’s care (88% positive ratings; *n* = 374) (Table 3). Parents who reported being offered options gave higher ratings compared to parents who did not for: involving parents in discussions about care (99% vs. 94% positive ratings, *p* = 0.01), eliciting information about their child (96% vs. 91% positive ratings, *p* = 0.04), and encouraging parents to provide suggestions for care (91% vs. 82% positive ratings, *p* = 0.04). There were no statistically significant differences in ratings between parents from ambulatory care and the emergency department.

**Table 3:** Ratings of healthcare teams’ efforts to engage parents in the decision making process

Items	Very poor *n* (%)	Poor *n* (%)	Fair *n* (%)	Good *n* (%)	Very Good *n* (%)
Involved parents (*n* = 408)	3 (0.7%)	0 (0%)	9 (2.0%)	86 (21%)	314 (76%)
Elicited parents’ knowledge (*n* = 383)	1 (0.3%)	2 (0.5%)	22 (6.0%)	129 (33%)	232 (60%)
Encouraged parents’ suggestions for care (*n* = 371)	7 (2.0%)	5 (1.0%)	32 (9.0%)	122 (33%)	208 (56%)

## Discussion

We explored parental perceptions of decision making involvement and decisional conflict. Overall, we found that nearly half of surveyed parents reported not being offered treatment options and almost a quarter screened positive for decisional conflict. Parents who reported being offered treatment choices were less likely to experience decisional conflict and more likely to understand the risks and benefits of their treatment decision, compared to parents who reported not being given options. Furthermore, parents that were offered options were nearly twice as likely to understand the information compared to those who did not perceive being offered options. More parents in the emergency department experienced decisional conflict compared to those from ambulatory care. Most parents provided positive ratings of the healthcare teams’ efforts to include them in the decision making process. Our results lead us to make four main observations.

First, many parents in our sample were insufficiently engaged from the first step of the decision making process (i.e., treatment options not identified by healthcare team), suggesting that their decisional needs were unmet immediately after their clinical encounter. These findings are consistent with literature reviews examining parental involvement in decisions about their child’s health [7, 8]. Indeed, a systematic review that specifically examined parental decision making needs showed that parents require good quality information (e.g., available options, risks and benefits) to make informed decisions on behalf of their child [34]. Good quality information can be obtained through shared decision making [16]. Our results also suggest that parents who are involved in the decision making process are less likely to experience decisional conflict. Another systematic review, which examined shared decision making interventions in pediatrics, found that parents had improved knowledge and less decisional conflict when engaged in shared decision making interventions [21].

Second, our study found that parents who perceived being provided options were more likely to: feel sure about the decision, understand the information, be clear about the risks and benefits, and have sufficient support and advice to make a choice. Our large sample size likely impacted the statistical significance of these findings. For example, the largest effect was for understanding the information, with a statistically significant absolute difference of 46% between groups. However, the absolute differences for other SURE test items were smaller (i.e., ranged from 7 to 11%), yet statistically significant. Moreover, the positive outcomes for these three items were already high (>85%). This creates challenges for interpreting the clinical significance of the findings. Currently, we are unaware of an accepted cut-off for determining the clinical significance of SURE test subscale items. Additional research is needed to determine criteria for interpreting the clinical significance of individual SURE tests items.

Third, we were not surprised that different decisional conflict rates were observed between ambulatory care and the emergency department. Previous studies have also found differences in decisional conflict across contexts [22–24, 33]. There are several potential explanations for these differences in parental decisional conflict. Parents from ambulatory care may have pre-existing relationships with healthcare professionals and are more likely to discuss treatment decisions along a continuum (e.g., management of chronic disease) [35]. In contrast, parents from the emergency department are more likely to have a first encounter with a particular healthcare professional and make decisions related to an acute issue, which may contribute to decisional conflict immediately after the consultation. The perceived urgency of decision making might differ across these contexts, influencing decisional conflict [36, 37]. Despite contextual differences, use of shared decision making in the emergency department and ambulatory care clinics has potential to improve outcomes. A systematic review showed that shared decision making in pediatric emergency departments is feasible and might improve parents’ knowledge, satisfaction, engagement, and help them clarify their values regarding treatment options [38]. A pilot study evaluating a shared decision making intervention (i.e., patient decision aid and decision coaching) in an ambulatory care clinic showed that parents and children who were early in the decision making process had reduced decisional conflict and thought that the intervention was acceptable [30]. Shared decision making interventions were also found to reduce parental decisional conflict when deciding about treatment for newly diagnosed attention deficit/hyperactivity disorder [39].

Fourth, parents who reported not being offered options still gave their healthcare team positive ratings for their efforts to involve them in the decision making process, albeit lower than those who perceived being provided options. For example, of the 48% of parents without options, 94% gave their healthcare team positive ratings for involving them in the decision making discussions. Although we were surprised by these findings, there are several potential explanations. It is possible that parents did not perceive presentation of options as a prerequisite of decision making involvement. Or, parents might have low expectations of healthcare professionals’ responsibility to engage them, thus providing a high rating when any effort to engage them is perceived. Also, parents’ preferred decision making roles and expectations for inclusion may depend on the child’s diagnosis, parents’ previous knowledge, socioeconomic status, and health literacy [7, 40–43]. Alternatively, parents’ might have interpreted the healthcare team ratings as an expression of satisfaction with their clinical encounter. If so, a ceiling effect whereby satisfaction with usual care, or the quality of the interaction, is already high might have influenced results; a phenomenon described in the adult patient decision making literature [16]. Nonetheless, satisfaction with the interaction or perceived involvement in the decision making process alone might not lead to high quality health decisions that are informed and consistent with patient and parent values if patients and family members are inadequately engaged in the process and experiencing decisional conflict.

The results of our study should be interpreted within the context and its limitations. The generalizability of our study findings is restricted for two reasons. First, we focused on two clinical settings within one pediatric hospital. Second, we collected limited demographic information from parents in favor of a short (1-page) survey that parents could complete quickly while attending a consultation with their child. Decisional conflict, however, has been shown to disproportionately affect more vulnerable populations (e.g., lower educational and racial and ethnic minorities) such as parents making decisions about their child with a life limiting disease [40, 44]. Further, our response rates were moderate and selection bias may have influenced our results if parents who enrolled differed from those who opted not to participate. We speculate that our response rates were also impacted by the unit receptionists’ availability and willingness to help with the study, highlighting the need to ensure study buy-in from all personnel before study implementation. Importantly, descriptive research aims to describe and explain situations and cannot determine causal relationships between variables [45]. Alternatively, descriptive research provides a foundation for specific hypothesis testing using experimental research methods.

## Conclusions

Almost half of parents in our study perceived they were not provided treatment options when discussing a health decision about their child. Presentation of options is critical for patient engagement and informed consent. Further, nearly a quarter of parents screened positive for decisional conflict immediately after their clinical encounter. Parents who perceived being offered choices were less likely to experience decisional conflict. Shared decision making is a promising intervention to improve parental involvement and reduce decisional conflict, however, more research evaluating its efficacy in the pediatric setting is needed.

## Additional file

Additional file 1: Decision Making Survey for Families. (DOC 55 kb)
