# Supplementary material for: Parental decision making involvement and decisional conflict: a descriptive study
Source: BMC Pediatr. 2017 Jun 13;17:146. doi: 10.1186/s12887-017-0899-4 (PMC5470309; doi:10.1186/s12887-017-0899-4)
Supplement: Additional file 1: — Decision Making Survey for Families. (DOC 55 kb) [file 12887_2017_899_MOESM1_ESM.doc]

**Please answer the questions below to help us learn how families participate in decisions about their child/youth’s health.**

**During your visit to CHEO today, did you discuss (i.e., make a decision about) any of the following topics?** Check all that apply.

❑ Tests (e.g. whether to have an x-ray, blood work, scan or procedure)

❑ Medications (e.g. whether to start a new medication, stop or change a medication)

❑ Surgery (e.g. whether to have surgery, wait or not have surgery)

❑ Follow-up with health professionals (e.g., whether to go and see a nurse, dietician, or other specialist)

❑ Behavioural intervention (e.g., change a behaviour such as diet, exercise, sleep)

**If not, then you do not need to complete this survey.**

**Which CHEO healthcare professional did you discuss this topic with?**

❑ Staff Physician ❑ Resident/Fellow ❑ Medical Student ❑ Nurse Practitioner

- Other

**Were you asked to consider more than one option?** ❑ Yes ❑ No

|  | **Sure of myself**  **Do you feel SURE about the best choice for you?** | Yes  ❑ | No  ❑ |
| --- | --- | --- | --- |
|  | **Understand information**  **Do you know the benefits and risks of each option?** | Yes  ❑ | No  ❑ |
|  | **Risk-benefit ratio**  **Are you clear about which benefits and risks matter most to you?** | Yes  ❑ | No  ❑ |
|  | **Encouragement**  **Do you have enough support and advice to make a choice?** | Yes  ❑ | No  ❑ |

| **Rate how CHEO's healthcare team handled the following:** | **Very Poor** | **Poor** | **Fair** | **Good** | **Very Good** |
| --- | --- | --- | --- | --- | --- |
| **Involved the parents/guardians in the discussion?** | ❑ | ❑ | ❑ | ❑ | ❑ |
| **Invited your** **family to share your knowledge/experience of your child/youth?** | ❑ | ❑ | ❑ | ❑ | ❑ |
| **Invited your family to share suggestions for care of your child/youth?** | ❑ | ❑ | ❑ | ❑ | ❑ |

**How old is the child/youth?**  years

**Who filled out this survey?** ❑ Mother ❑ Father ❑ Other
